# Complete genome sequence of a nitrogen-fixing bacterium, *Klebsiella pasteurii* (formerly *Klebsiella oxytoca*) NG13, a rice rhizosphere isolate

**DOI:** 10.1128/mra.00885-24

**Published:** 2024-11-14

**Authors:** Daisuke Yoshidome, Makoto Hidaka, Saori Kosono, Makoto Nishiyama

**Affiliations:** 1 Graduate School of Agricultural and Life Sciences, The University of Tokyo, Bunkyo-ku, Tokyo, Japan; 2 Kikkoman Corporation, Noda, Chiba, Japan; 3 Collaborative Research Institute for Innovative Microbiology, The University of Tokyo, Bunkyo-ku, Tokyo, Japan; 4 RIKEN Center for Sustainable Resource Science, Wako, Saitama, Japan; University of Strathclyde, Glasgow, United Kingdom

**Keywords:** *Klebsiella oxytoca*, rice rhizosphere, complete genome sequence, nitrogen fixation

## Abstract

*Klebsiella oxytoca* NG13 was isolated from the rice rhizosphere in the early 1980s, and its nitrogen-fixing ability has been investigated. For the further advanced research, we sequenced its genome. NG13 contains one chromosome of 5,897,538 bp and no plasmid. NG13 should be classified to *Klebsiella pasteurii* based on ANI.

## ANNOUNCEMENT

*Klebsiella oxytoca* NG13 was isolated in the early 1980s through a project to make an inventory of free-living diazotrophs in the rhizosphere of rice grown in a paddy field (Mishima, Shizuoka, Japan) of the National Institute of Genetics, Japan (1, 2). Since then, we have studied the nitrogen-fixing ability of NG13 (3–5). Current genome-based taxonomic studies have shown that *K. oxytoca* is not a single species but a complex (*K. oxytoca* complex) of at least six species: *Klebsiella grimontii*, *Klebsiella huaxiensis*, *Klebsiella michiganensis*, *K. oxytoca*, *Klebsiella pasteurii*, and *Klebsiella spallanzanii* (6). In this study, we determined the complete genome sequence of NG13 for further advanced research.

NG13 was provided by Dr. Komagata, an author of References 1 and 2, immediately after the isolation, and Hidaka has managed it for about 40 years as a 30% glycerol stock at −80°C. We recently deposited it with the National Institute of Technology and Evaluation, Japan, as NITE P-03721.

A single colony of NG13 from the glycerol stock was inoculated to LB liquid medium (Thermo Fisher Scientific, Tokyo, Japan), cultured for 18 h at 30°C, and its genomic DNA was extracted by Wizard genomic DNA purification kit (Promega, WI, USA). The sequencing process was conducted by the Bioengineering Lab. Co., Ltd. (Kanagawa, Japan) as follows. The extracted DNA was fragmented to approximately 10–20 kb by the g-TUBE (Covaris, MA, USA). A library was constructed using the SMRTbell prep kit 3.0 (PacBio, CA, USA) and SMRTbell gDNA sample amplification kit (PacBio). The library was treated with the Revio polymerase kit (PacBio) and sequenced using the Revio (PacBio). 27,315 High-fidelity (HiFi) reads (average length 9,084 bp) with quality values greater than 20 were obtained. 23,160 HiFi reads of >1,000 bp were assembled, and only 1 circular contig was obtained by using Flye (ver. 2.9.3-b1797) (7). CheckM2 (ver. 1.0.1) (8) revealed completeness of 100% with genome coverage of 42.1-fold. Default parameters were used for all software. NG13 has a single chromosome of 5,897,538 bp with a G+C content of 55.4% and no plasmid. The sequence was not rotated to a specific start gene.

The gene annotation and taxonomy check based on average nucleotide identity (ANI) of the NG13 chromosome sequence were performed by us using DFAST v1.6.0 provided by DDBJ (9) using default parameters. The chromosome is predicted to contain 5,406 protein-coding genes, 87 tRNAs, and 25 rRNA. Among the ANI values of NG13 chromosome to chromosomes of the type strains of six species of *K. oxytoca* complex, the highest one was 99.32% to *K. pasteurii* (accession number GCA_902158725.1), and the ANI value (91.53%) to *K. oxytoca* (accession number GCA_020115535.1) was fourth out of six. These suggest that NG13 should be classified to *K. pasteurii* rather than *K. oxytoca*.

## Data Availability

The complete genome sequence of *K. pasteurii* NG13 has been submitted to DDBJ/ENA/GenBank under the accession number AP035775. The sequencing data can be found in the DDBJ Sequence Read Archive under the accession number DRR576807.
